# Neural architectures in the light of comparative connectomics

**DOI:** 10.1016/j.conb.2021.10.006

**Published:** 2021-12

**Authors:** Elizabeth Barsotti, Ana Correia, Albert Cardona

**Affiliations:** 1 MRC Laboratory of Molecular Biology, Cambridge, UK; 2 Department of Physiology, Development and Neuroscience, University of Cambridge, UK

## Abstract

Since the Cambrian, animals diversified from a few body forms or *bauplans*, into many extinct and all extant species. A characteristic neural architecture serves each *bauplan*. How the connectome of each animal differs from that of closely related species or whether it converged into an optimal architecture shared with more distant ones is unknown. Recent technological innovations in molecular biology, microscopy, digital data storage and processing, and computational neuroscience have lowered the barriers for whole-brain connectomics. Comparative connectomics of suitable, relatively small, representative species across the phylogenetic tree can infer the archetypal neural architecture of each *bauplan* and identify any circuits that possibly converged onto a shared and potentially optimal, structure.

## Highlights

• Technological innovations have lowered the barriers for whole-brain connectomics.
• Comparative connectomics (CC) across suitable representative species is now feasible.
• CC infers the common neural architecture across closely related animal species.
• CC reveals the neural circuit details that make animals different from each other.
• CC identifies structurally convergent circuits in distantly related species.

## Introduction

**Figure 1:**
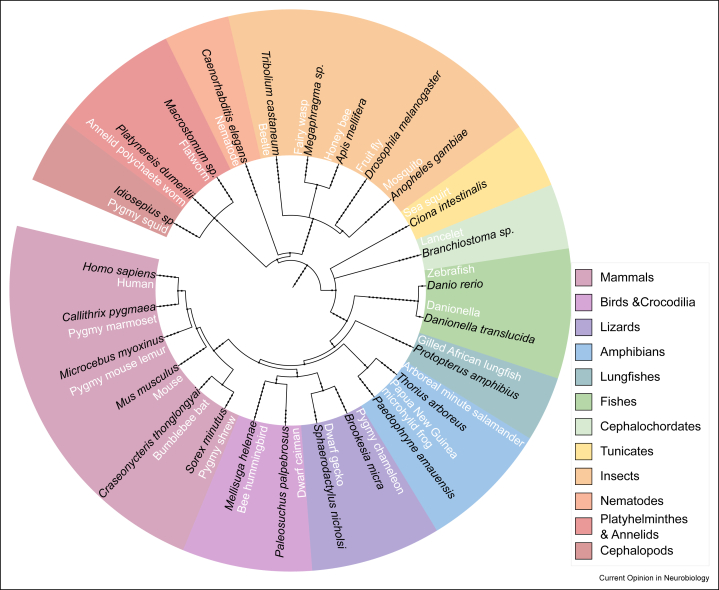
Phylogenetic tree of possible candidate reference species for comparative connectomics plus a few others for reference such as humans. See also Table 1.

Five hundred and fifty million years of evolution have generated an astounding diversity of animal forms with unique neural architectures. Grouped by common descent, each animal clade presents a characteristic, identifiable *bauplan*, served by an idiosyncratic neural architecture. While similarities are more evident among recently diverged groups, deep homologies among distant clades have been suggested by gene expression [1], developmental patterns [2], and neural organization [3]. In vertebrates, the division of the pallium into four neurogenetic regions has been reported in clades as distant as fishes and mammals [4]. In invertebrates, neuroblasts and their progeny of neuronal cell types are conserved not only across insect orders [5,6] but also within outgroups, such as crustaceans [7] and tardigrades [8]. With insects [9], fishes [10], and cephalopods [11] dating from the Cambrian, their nervous system architectures have remained stable over hundreds of millions of years while simultaneously specializing in a myriad of ways under evolutionary pressures. An approach to analyze neural architectures is comparative connectomics, consisting of first mapping the synaptic wiring diagram or connectome, of the whole or a part of a nervous system, and then analyzing its structure relative to another connectome, either of another species or of a different genotype or life stage [12∗∗, 13∗∗, 14∗∗]. A comparative connectomics approach across representative species (Figure 1) would overcome inter- and intraspecies variability to identify the fundamental neural circuit architecture of each *bauplan* as well as any shared yet nonhomologous circuit, a product of convergent evolution.

**Table 1:**
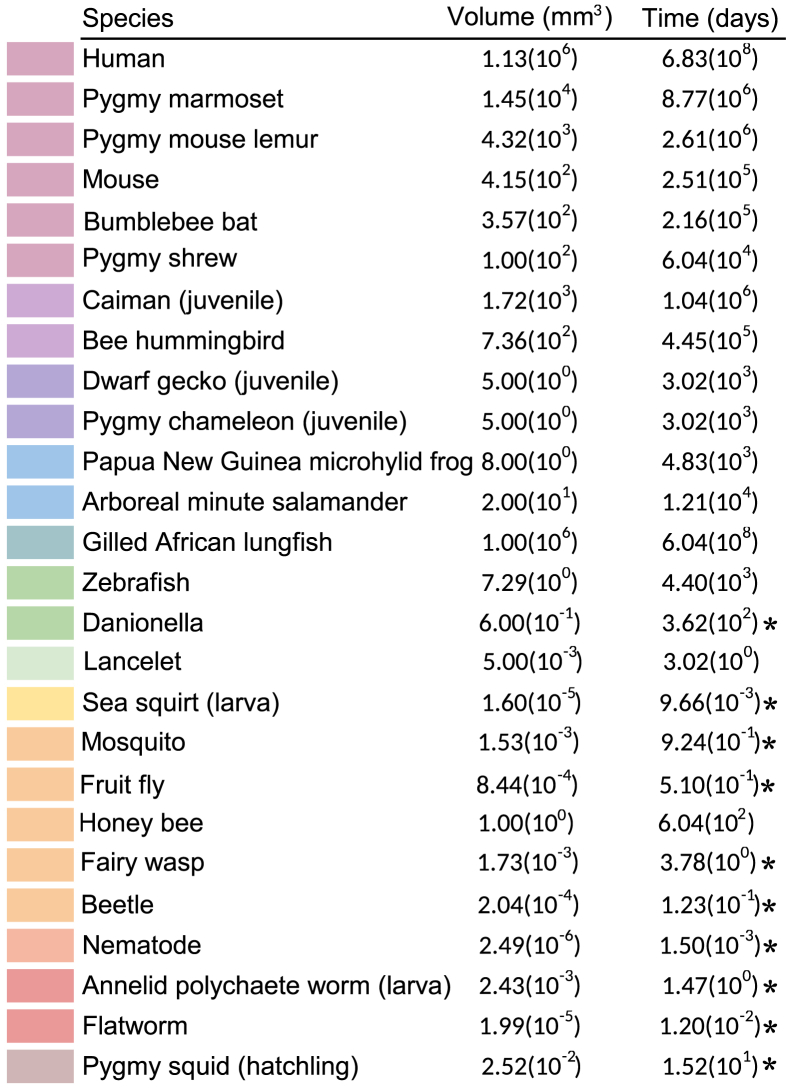
List of brain volumes and estimated imaging time with GridTape TEM [38], using the formula *Time*_*imaging*_ = (*Volume*_*brain*_∗151/0.25), with 151 being the number of days necessary to acquire a volume of 1×0.5 × 0.5 mm according to Graham et al. (2019). Asterisk, volumes that can be acquired in less than or up to about a year of 24/7 imaging. TEM, transmission electron microscopy.

## Existing connectomes

Today, connectomics research focuses primarily on the mouse [15, 16, 17∗∗], fruit fly [18, 19∗, 20, 21∗, 22∗], nematode *Caenorhabditis elegans* [14,23, 24, 25], and zebrafish [26], with additional contributions from polychaete worms (*Platynereis* sp.; [27]), chordates (*Ciona intestinalis*, [28]), and others. This contrasts with the origin of neuroscience as a discipline, where more and diverse species were used [29,30], leading to seminal discoveries such as action potentials in the giant axon of the squid [31], synaptic function in *Aplysia* [32], and in the relationship between neural circuit structure and function in crayfish [33,34].

For the four species that dominate neuroscience research, there is a complete connectome for one (*C. elegans* [14,23, 24, 25]) as well as complete electron microscopy (EM) volumes with partial connectomes for two (*Drosophila* [18, 19∗, 20, 21∗, 22∗] and *Danio rerio* [26]) and a proposal to map one (*Mus musculus* [35]). Broadening our reach beyond these few species will open up the opportunity to discover fundamental neural circuit architectures [30,36].

## Techniques

Step improvements in electron microscopy (EM) automation, namely reliable focused ion beam scanning electron microscopy (FIB-SEM) for isotropic small volumes [37] and GridTape transmission electron microscopy (TEM) for much larger volumes [38], have expanded the set of feasible species (Table 1) to larger animals. Such larger samples require reliable, high-performance image registration methods to assemble continuous EM volumes from millions of image tiles, overcoming nonlinear deformations and artifacts [39,40]. In turn, larger EM volumes have shifted the focus from manual methods for neuronal arbor reconstruction and synapse annotation [41,42,43] to automated ones that target precision (accuracy) at the expense of recall (completeness) [21,44, 45, 46], guided by studies on redundancy in synaptic connectivity [42,47]. Reconstructed neurons are then matched across image modalities by registration and morphological similarity (e.g. with neuron BLAST (NBLAST); [48]), enriching connectomes with functional information [15, 16, 17∗∗, 18] or neurotransmitter signatures [18], with the latter also inferred directly from EM [49]. Comparing the resulting connectomes across left-right symmetric brain hemispheres or across individuals or species requires matching graph nodes — where a node is a neuron or a group of neurons in a coarsened graph — by either exploiting known shared molecular information, location, and morphology (such as insect neuroblasts and their progeny of neurons [5,6] or cortical neurons [50]) to seed the alignment of at least some nodes across graphs [51] or from connectivity only with spectral graph analysis [24,42]. With improvements across the board, we now have the opportunity to traverse the whole phylogenetic tree to sample representative species in the light of comparative connectomics.

## Suitable representative species

An ideal data set includes whole-brain connectomes of both closely related and widely divergent species. Presently, only EM of densely labeled samples can deliver the complete, nanometer-resolution volumes necessary for mapping every neuronal arbor and synapse. A number of practical constraints reduce the pool of possible species (Table 1).

First, sample preparation for densely labeled, whole-brain connectomics is lengthy and costly, as evidenced by work in *Drosophila* [52] and the mouse [53]. Small brains available in large numbers ease the development of sample preparation protocols, which favor animals with fast life cycles and abundant progeny.

Second, in practice, sample dimensions are constrained to ∼1 mm^3^ by the combination of resolution requirements, imaging speed, data costs, and funding cycles.

Third, free-living, nonparasitic small animals retaining a full complement of ancestral body parts and brain structures are best suited for both comparisons of individual brain modules and whole-brain architectural relationships. As follows, the comparison of a blind fish, for example, with an anosmic snake would be limited to brain structures besides olfaction and limb-based locomotion. This constraint favors a small lizard [54, 55] over a small soil-dwelling anosmic snake that presents poor vision [56] and, likewise, favors a small nonblind fish, such as *Danionella* sp. [57]. Exceptionally, species that lost body parts while retaining the corresponding neural modules would serve as models for how a neural architecture takes on new functions (e.g. visual inputs dominate the mushroom bodies of anosmic beetles [58]), a situation inducible experimentally [59, 60, 61∗∗].

To overcome most constraints, an option is to consider juveniles. Typically, some animal groups present juveniles that closely resemble adults, such as coleoid cephalopods, reptiles, and some fishes, among many others. This approach works best when juveniles live independently of parental care, indicating that all aspects of their brains are already functional, except for those related to sexual maturity. An example, if unconstrained by dimensions would be the juvenile of some crocodiles that have been shown to present approximately the same number of neurons and presumably the same overall neural architecture, as the adults, differing primarily in neuronal cell size, not number [62]. Within the dimensional constraints, we find free-living lizard hatchlings, such as the chameleon *Brookesia* sp. [54] and the gecko *Sphaerodactylus* sp. [55, 63∗], and cephalopod hatchlings, such as *Idiosepius* sp. [64]. A comparative connectomics approach targeting free-living juveniles would save time and resources (animal length ∼ volume^3^) while meeting the above constraints and compromising only on circuits associated with sexual maturity.

## Case studies

### Evolution of a brain structure: the cortical microcircuit

The apparent uniformity of the mammalian neocortex [65] suggested the existence of a basic microcircuit repeated throughout all cortical areas [66]. On the basis of sparsely sampled neuronal anatomy and electrophysiology of the cat and monkey visual cortex, a diagram for the basic cortical microcircuit was proposed, limited to excitatory neurons [67]. The addition of inhibitory connections led to the reformulation of the diagram as a canonical microcircuit that captured commonly observed motifs across multiple areas and species and which suggested fundamental features of cortical processing [68]. Mainly, the inseparability of excitation and inhibition, and the primacy of intracortical excitation over thalamic drive. Synaptic weights were later estimated from further sparse anatomical reconstructions [69].

**Figure 2:**
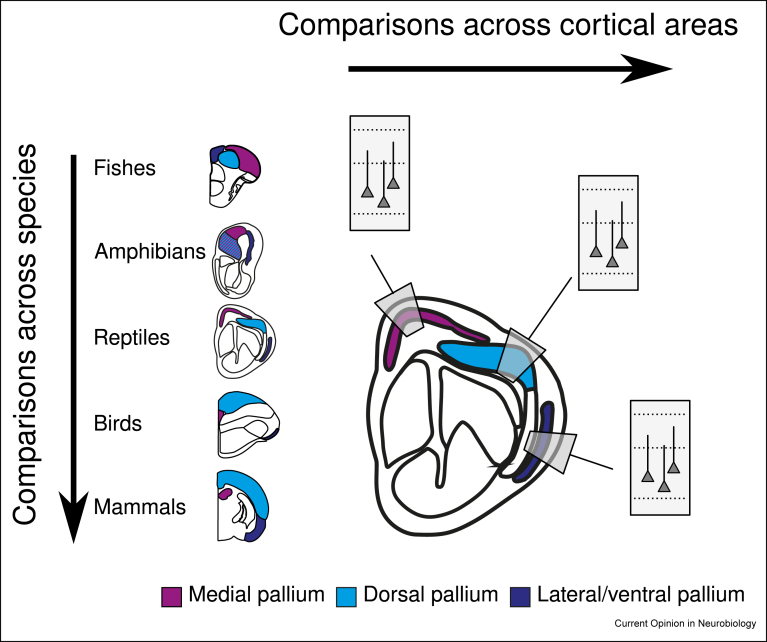
Schematic representation of the comparison of cortical microcircuits across brain areas and species, on the basis of known genetic and developmental correspondences within the vertebrates. Cartoons show the right brain hemisphere; adapted from Naumann et al., 2015, with permission.

The hypothesis of a repeated unit of computation throughout the cortex is attractive for its reductionist properties: the study of the immense cortical sheet becomes the study of its building block and interblock relationships. Dense reconstructions of volumes of cortex at synaptic resolution possibly containing a complete canonical microcircuit have only recently become possible for limited subregions of the mouse brain [15, 16, 17∗∗]. While the many similarities in microcircuit structure across cortical areas grant enormous support to the canonical microcircuit hypothesis, differences exist across areas and species [70, 71, 72, 73, 74, 75]. The reconstruction of multiple instances of the cortical microcircuit in multiple cortical areas of various vertebrates will identify commonalities and differences in the cortical microcircuit of each brain area and species (Figure 2).

All amniotes — mammals, birds, and the polyphyletic reptiles — present a layered cortical sheet suitable for the study of the homogeneity or heterogeneity of the canonical microcircuit [76,77]. The architecture of the vertebrate forebrain is thought to be conserved across all vertebrates, including the lamprey [78, 79].

Extant reptiles offer a useful model of vertebrate cortical architectures [50]. Juvenile lizards, including *Brookesia* sp. [54] and *Sphaerodactylus* sp. [55] [63], are free-living predators with a complete tetrapod *bauplan* and brain volumes of ∼1 cubic millimeter.

Comparative connectomics of cortical columns from the same homologous brain regions across species, such as lizards and rodents, will highlight a possibly conserved cortical microcircuit and which circuit motifs are unique to mammals. Across the whole brain, such comparisons will further identify large-scale common circuits interrelating different cortical areas and whether such patterns are already present outside amniotes. In summary, mapping the cortical architectures of multiple small vertebrates opens the opportunity to infer the archetypal cerebral circuit architecture.

### Evolution of a substrate for computation: circuits for pursuit predation

Vision-driven behaviors, such as pursuit predation (the tracking and interception of prey), are present in coleoid cephalopods, vertebrates, and insects. Successful predation requires the integration of the prey motion vector with self-motion to intercept the prey [80]. Supporting these abilities is a visual system capable of distinguishing prey from background, tracking prey relative motion, and anticipating future prey position. Although coleoid cephalopods, vertebrates, and insects contain vastly diverse nervous systems, all comprise species that engage in pursuit predation. The possibility exists that some aspects of the neural circuit architectures for visually guided predation have converged throughout evolution into a common, optimal configuration in animals with shared ecological niches such as fishes and squids.

Pursuit predation consists of three major components: visual tracking of prey; computation of speed and direction vectors; and motor planning toward interception.

**Figure 3:**
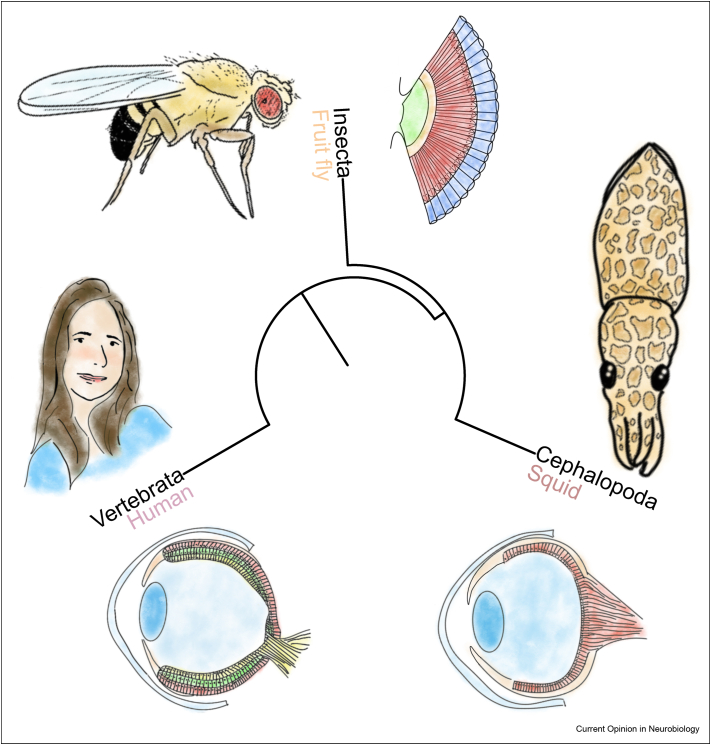
Phylogenetic tree illustrating differences and similarities in eye structure among insects, coleoid cephalopods, and vertebrates, with the insect presenting a compound eye and the other two a camera eye. For full comparisons with the multilayered circuits of the vertebrate retina, additional brain structures of the insect and cephalopod must be considered such as their corresponding optic lobes. Blue, lens or crystalline cone; red, photoreceptors. Original hand drawings by Ana Correia.

Vertebrates and coleoid cephalopods present camera eyes of superficial similarities but deep developmental and structural differences [81], whereas insects present compound eyes (Figure 3). Despite divergent eye morphology, strong parallels have been found in the circuits for motion detection in mammals and insects [82]. The coleoid cephalopod's visual circuits are mostly unknown but present a single-layer retina and an associated optic lobe [83]. Visual signals in all three animal groups arrive at the brain already processed: in mammals by the multilayered retina; and in insects and cephalopods by the optic lobes. The early visual circuits of the retina or associated optic lobes compute direction of motion of objects in the visual field, in insects and mammals [82]. Presumably, the visual circuits of coleoid cephalopods, like the fly's, also implement an equivalent to the Hassenstein-Reichardt motion detector [84]. The mammalian and insect retinas have been studied in depth with connectomics [84, 85, 86, 87]. Comparative connectomics of the visual circuits of species that share a *bauplan* (e.g., squids and octopus) will establish a baseline against which any similarities with species of other *bauplans* (e.g., insects and mice) could be interpreted as potentially optimal, products of convergent evolution.

In pursuit predation, in addition to tracking prey motion, the predator must compute an intersection trajectory that not only compensates for prey motion but also accounts for its own head and body motion. In mammals, circuits in the midbrain including the superior colliculus represent motor space [88]. In insects, circuits in the central brain including the central complex encode body direction [89]. The geometric computations of the multiple direction vectors are implemented in neural circuits whose architecture can be compared across species. Whether the circuits for spatial orientation share an overall architecture across species can be studied by whole-brain comparative connectomics of suitably small species.

All foraging animals, regardless of limb presence and overall body morphology, are endowed with different locomotion modes; therefore, the neural circuits for coordinated movement postsynaptic to command neurons will be idiosyncratic for each. Upstream, in neural circuits for decision making, surprising conservation has been observed. One remarkable example of conserved intermediate circuits connected to different, specialized motor modules is the Mooncrawler/Moonwalker neuron, which controls backward locomotion for both the limbless *Drosophila* larva and the legged adult [90]. In analyzing the connectomes of vertebrates, insects, and coleoid cephalopods, we expect extreme diversity of neural circuits for locomotion but potentially shared neural architectures for optimally computing direction vectors and behavior selection.

The connectomes of *Drosophila*, the zebrafish, and the mouse retina are either complete or imminent, whereas no studies have yet mapped the visual circuits of a cephalopod. Meeting all of the constraints, the pygmy squid *Idiosepius* sp. and the pygmy octopus *Octopus joubini* both present free-living juveniles with brain volumes within a cubic millimeter. Mapping the cephalopod connectome from an EM volume of the whole body, as is now possible for *Idiosepius* juveniles, will address further questions central to this phylum, including camouflage control [91] and soft limb coordination throughout multiple styles of locomotion and tool manipulation.

### Analysis of diversification: the insect brain

Insects are likely the most speciose group of animals on Earth, with the Coleoptera (beetles; particularly the Phytophaga clade [92]), Hymenoptera (wasps, bees, ants, and sawflies; particularly parasitoid wasps [93]), and Diptera (flies, midgets, and mosquitoes; particularly the Cecidomyiidae family [94]) being extraordinarily rich. The impact of insects on human life is immense, either as pests or vectors of deadly diseases (mosquitoes; [95,96]) or for their vital ecosystem services, such as the pollination of crops and pest control [97]. An approach to pest control that harms pollinators would result in a net loss, a predicament human societies are currently facing. Comparative connectomics would reveal the commonalities and particularities of each insect group and enable the design of targeted pest control, for example, by molecularly targeting circuits for human-seeking behavior (e.g. CO2 plume tracking [98]) while avoiding interference with beneficial services such as pollination (e.g. sensing flower-specific odors [99]).

Beyond the use of insects as experimental subjects for understanding cognition [100,101], these tiny animals pack mighty abilities, rivaling computer vision systems many orders of magnitude their size with extremely small energy requirements, and have been a continuous source of inspiration in engineering (e.g. [102, 103∗, 104∗]) and machine learning (e.g. [104∗, 105∗]). The reduced dimensions and numerically reduced nervous systems of insects offer unmatched experimental tractability.

The connectomes of the adult and larval *Drosophila* brain are almost fully mapped [18, 19∗, 20,22]. Among the Hymenoptera, species as large as bumblebees [106] and as small as fairy wasps [107] are currently under study. Meeting our criteria of small, complete, free-living, accessible species, we find the adult *Drosophila melanogaster*, the beetle *Tribolium castaneum*, the honeybee *Apis mellifera*, and the mosquito *Anopheles gambiae,* and all four of them are already laboratory animals and realistic targets for whole-brain connectomics today. The brains and nerve cords of all of these species share a recognizable architecture, with genetically identified neuroblasts [5,6] and individual neurons morphologically recognizable across species. Comparative connectomics across these species would produce an approximated insect brain neural circuit archetype, alongside species-specific brain modules and circuit motifs that confer each insect group with unique properties. With these animals, we now have the opportunity to understand in what way each different insect species has specialized its brain to better fit its ecological niche, in a process of divergent evolution, and how, in a process of convergent evolution, some of their brain structures — for example, the olfactory system [108,109], the visual system [82], and the learning and memory system [110] — have converged with those of distantly related animals.

## Conclusion

The study of neural circuit architectures with synaptic resolution, or connectomics, has until now focused on a few species, primarily a nematode, a fly, a fish, and a mouse. Concentrating resources on few species generated synergies from the sharing of tools, databases, and understanding, which carried the neuroscience field forward. Now, technological improvements across the board open the opportunity to explore the diversity of nervous systems across the tree of life. With comparative connectomics, the search for neural circuit architectures common across species or independently converged into an optimal layout is now possible.

## Conflict of interest statement

Nothing declared.
